# Grief in Neurosurgery: Reframing Loss and Identity Through Lived Experience

**DOI:** 10.1227/neuprac.0000000000000294

**Published:** 2026-09-16

**Authors:** Erin N. Walker, Vidhatri Raturi, Kathleen S. Botterbush, Anna R. Kimata, Sangami Pugazenthi, Megan M. J. Bauman, Ian Parney, Kimberly Kicielinski, Karin Muraszko, Violette Recinos, Shelly Timmons, Laura Snyder, Susan C. Pannullo, Rupa Juthani, Angela M. Richardson, Maryam Rahman

**Affiliations:** 1 University of South Carolina School of Medicine Greenville, Greenville, South Carolina, USA;; 2 Tulane University School of Medicine, New Orleans, Louisiana, USA;; 3 St. Louis University School of Medicine, St. Louis, Missouri, USA;; 4 Warren Alpert Medical School of Brown University, Providence, Rhode Island, USA;; 5 Department of Neurosurgery, University of Pittsburgh Medical Center, Pittsburgh, Pennsylvania, USA;; 6 Department of Neurosurgery, Washington University, St. Louis, Missouri, USA;; 7 Department of Neurosurgery, Mayo Clinic, Rochester, Michigan, USA;; 8 Department of Neurosurgery, Medical University of South Carolina, Charleston, South Carolina, USA;; 9 Department of Neurosurgery, University of Michigan, Ann Arbor, Michigan, USA;; 10 Department of Neurosurgery, Cleveland Clinic, Cleveland, Ohio, USA;; 11 Department of Neurosurgery, Medical College of Wisconsin, Milwaukee, Wisconsin, USA;; 12 Department of Neurosurgery, Barrow Neurological Institute, Phoenix, Arizona, USA;; 13 Department of Neurological Surgery, Weill Cornell Medicine, New York, New York, USA;; 14 Department of Neurosurgery, NYU Langone Health, New York, New York, USA;; 15 Department of Neurosurgery, Indiana University Health, Indianapolis, Indiana, USA;; 16 Department of Neurosurgery, University of Florida, Gainesville, Florida, USA

**Keywords:** Challenges, Career, Family, Loss, Disability

## Abstract

Grief is an under-recognized yet pervasive experience in neurosurgery, arising not only from the death of patients and loved ones but also from losses related to professional identity, health, and personal relationships. Despite its relevance, grief is often conflated with burnout or overlooked entirely within surgical culture. When personal adversity strikes, such as loss, illness, or major life upheaval, the intensity of neurosurgical practice can amplify these challenges, influencing professional identity, performance, and overall resilience. As the field continues to evolve and diversify, it is increasingly important to foster open dialogue about vulnerability, personal hardship, and life beyond the operating room. This perspective examines how neurosurgeons experience and process loss, distinguishing grief from burnout and resilience, and highlighting the need for institutional and cultural change.

**ABBREVIATIONS:**
CNS: Congress of Neurological Surgeons.

Heartbreak, anxiety about the unknown, and having to navigate a new reality after loss are part of the human experience. Cataclysmic changes in life ask people to pull deep within themselves, finding resources such as resilience, patience, ingenuity, and flexibility to be able to endure life's challenges. Neurosurgeons are particularly well-dispositioned to weather the storms, given their ability to overcome obstacles in the pursuit of becoming a neurosurgeon.

Practicing neurosurgeons have typically overcome social barriers, proven themselves as high achieving in school, and often excel at activities outside of their education as well. As a profession, neurosurgery selects for people who accept delayed gratification, demonstrate toughness at work, and model professionalism even in the most stressful situations. Selection of these types of people is also associated with significant career satisfaction. Despite long hours and taking care of sick patients, 81% of US neurosurgeons report career satisfaction.^1^ The ability to perform in this high-stress, high-impact environment is a point of pride and identity for many neurosurgeons.

The triumphalism surrounding the neurosurgical profession is seen in marketing campaigns, articles about patients, and reports on research breakthroughs. However, this often obscures the reality of many people in neurosurgery. Many experience tragedy, divorce, loss of job, a new disability, or other significant life change. These experiences often significantly affect how neurosurgeons view their career, how they interact with patients, and what they prioritize on a daily basis. Because these life changes are not at the forefront of discussion in neurosurgical meetings and publications, the American Association of Neurological Surgeons/Congress of Neurological Surgeons (CNS) Section on Women in Neurosurgery hosted a session at the 2024 CNS Annual meeting entitled “Love, Life, Loss and Chaos in Neurosurgery” to address these issues and how leaders who have experienced personal crisis in this field have found balance and resilience.

The objective of this article was to summarize the lessons learned from this session in tandem with discussion of primary literature regarding how crisis has affected home life and work, strategies to find meaning despite difficulties, and the personal growth that takes place afterward. To address these topics, we divide the article into 2 overarching themes: professional and personal hardship, where subtopics of work constraints, divorce, disability, family planning, and personal loss can be explored more thoroughly. The purpose of this article was to bring to light the hardships that are experienced by all neurosurgeons and how often these can be more meaningful in a surgeons development compared with the victories. The victories are often what finds their way into social media posts, articles, news stories, and clips. However, it is the ability to come back to the OR and clinic after experiencing tragedy that often makes surgeons better equipped to care for themselves, their families, and their patients.

## GRIEF, BURNOUT, AND RESILIENCE

Grief, defined as the psychological, emotional, and behavioral response to loss, is distinct from but often conflated with burnout, a syndrome characterized by emotional exhaustion, depersonalization, and reduced personal accomplishment. While burnout has been extensively studied in neurosurgery, grief remains underexplored, despite its relevance to both personal and professional experiences of loss. Failure to distinguish between these constructs may lead to an incomplete understanding of physician distress and inadequate support structures.

Studies on grief reveal that variation exists between the way men and women express grief, but there is not a difference in duration or trajectory of grief based on sex.^2^ However, the reception of support in a time of grief is significantly different between men and women. Men are reported to receive less support, despite the expression of depressive symptoms or loneliness during bereavement compared with women.^3^ This disparity underscores the importance of extending comparable levels of support to male colleagues that female colleagues are afforded during times of bereavement or prolonged grief.

## PROFESSIONAL CHALLENGES IN NEUROSURGERY

The neurosurgical profession is characterized by intense clinical demands and prolonged work hours, necessitating substantial personal and temporal investment. Personal life stresses rarely coincide with lighter schedules or planned time off. Instead, they tend to surface during already stretched schedules, when neurosurgeons are least equipped to recalibrate. Cognitive bandwidth narrows, concentration falters, and small mistakes begin to accumulate in ways that feel foreign to the usual standard of performance. There is evidence that recent personal stress reduces working memory and attention, both of which are essential to procedural accuracy and clinical decision making.^4^ Physicians managing significant personal stress have also been found to report higher rates of perceived medical errors and are more frequently named in malpractice claims.^5,6^ In neurosurgery, where precision and poise are essential, the cognitive and emotional weight of personal stress can affect performance in subtle ways. Because it is often masked by professionalism and may not present like conventional burnout, personal stress can be difficult to recognize until it begins to affect well-being or clinical work.

### Burnout

Many neurosurgeons, when reflecting on their formative education and training, identify a deep intrinsic satisfaction derived from task completion and achievement. This orientation toward productivity and mastery aptly captured by Ralph Waldo Emerson's observation that “the reward of a thing well done, is to have done it” resonates strongly within the field. Although this disposition supports excellence and perseverance, it also represents a nidus for tension when unpredictable life events are compounded onto the stress to strive for perfection. On one hand, the sacrifices inherent to neurosurgical training and practice cultivate resilience, discipline, and the capacity to tolerate delayed gratification. These attributes foster the development of “grit,” enabling individuals to endure sustained effort without immediate reward and to navigate uncertainty or unfavorable outcomes. On the other hand, the same professional demands may conflict with the time, emotional presence, and recuperative capacity required during periods of personal crisis, such as illness, bereavement, or major life transitions.

Within a professional culture where long hours and constant availability for emergencies are the standard, personal crises may be amplified by both internal and external expectations. Neurosurgeons facing events such as the death of a loved one, divorce, or the onset of disability must not only manage the direct psychological and logistical consequences of these experiences but also contend with perceived or actual reductions in professional performance. The cumulative burden of physical and psychological stressors, particularly when personal crises intersect with professional challenges, can ultimately manifest as burnout.

Like neurosurgery, the occurrence of professional burnout is observable in other high-performing careers such as professional athletes. Psychological studies of high-performance athletes describe the importance of early detection and implementing personalized interventions that incorporate mindfulness and gratitude as supportive mechanisms for burnout prevention and recovery at the individual level. Coaching staff are also encouraged to reduce systemic stressors and avoid inadequate recovery time to encourage the best performance from their athletes.^7^ These insights reveal that addressing professional burnout in high-performance fields requires a combination of individual proactive strategies, such as mindfulness, and systemic support to ensure sustained performance over the duration of a career.

### Compensations Models and Academic Productivity

In addition to the personal struggles at work in the setting of crisis, the pressures for productivity in most neurosurgical careers is increasing. Even most academic neurosurgery jobs follow a work relative value unit compensation model in which clinical work is incentivized.^8-10^ However, teaching and research expectations are maintained, thereby further compounding an untenable situation for a neurosurgeon in distress at home. Often, at times of personal crisis, the work that is directly compensated (surgery and clinic) is maintained but other efforts such as teaching, administrative tasks, and research are severely affected.^11^ This imbalance not only affects academic productivity and departmental contributions but can also strain collegial relationships and mentorship obligations. Over time, the cumulative effect of these disruptions may hinder career advancement, contribute to feelings of inadequacy, and exacerbate burnout in a specialty already known for its high emotional and physical demands.^1,12^

## PERSONAL CHALLENGES IN NEUROSURGERY

### Personal Disability

Disability among physicians is more prevalent than often acknowledged. A national survey found that approximately 3.1% of US physicians self-identified as having a disability. The most reported disabilities included chronic health conditions (30.1%), mobility impairments (28.4%), psychological conditions (14.2%), hearing impairments (12.1%), and visual impairments (7.8%).^13^ In surgical specialties, the physical demands of the profession contribute to a higher incidence of work-related musculoskeletal disorders. Surgeons experience elevated rates of degenerative cervical spine disease (17%), rotator cuff pathology (18%), degenerative lumbar spine disease (19%), and carpal tunnel syndrome (9%).^14,15^ These conditions can significantly affect a surgeon's ability to perform operative procedures and may lead to temporary or permanent disability. To reduce the risk of practice-related injury, neurosurgeons are encouraged to optimize ergonomics through proper positioning and appropriate use of instrumentation, utilization of gel mats and supportive footwear to reduce lower back strain, and the integration of optical and motorized technology to assist with visualization while maintaining neutral posture and reduced physical stress caused by repetitive motion.^16^

However, the true sudden development of a life-altering disability often results in neurosurgeons exiting practice.^17^ Due to this loss from the profession, opportunities to support neurosurgeons experiencing such a dramatic change are limited. We also do not hear of the heart break, the insecurity, and the fear that ensues after disability. One of our panelists shared the first moments of realization that they had lost the ability to walk. The immense fear associated with the initial realization is often a traumatic experience and can affect future decision making.

In a beautiful account of her own struggles with sudden blindness and becoming a triple amputee after severe sepsis during her second pregnancy, Carol Decker writes in her book Unshattered: “Even the smallest broken pieces of a life can be put back together.” Our panelist described her eventual recovery from the fear. One of her methods to overcome insecurity about taking care of patients in the OR was to perform mentored cases with colleagues to assess which procedures could be performed in the setting of the new disability, thereby rebuilding confidence and ensuring patient safety.

Despite these individual successes, systemic barriers persist. A cross-sectional cohort study of general surgery residents found that while 83.9% reported their disability affected their work, 62.9% had not disclosed their disability to program directors, often due to fear of discrimination.^18,19^ This underreporting emphasizes the need for more supportive and transparent environments that encourage disclosure and provide necessary accommodations. The push to make neurosurgery adaptable and accessible to surgeons of all physical abilities is only beginning but requires the awareness and cumulative efforts of neurosurgeons at all levels.

### Divorce

Despite increasing awareness and acknowledgement of the potential impacts of a surgical career on one's personal affairs, the divorce rate among both surgeons and surgical trainees remains higher than their nonsurgical colleagues. In a 2025 study, the divorce rate among surgeons was found to be 21.3%, relative to 17.9% in nonsurgeons.^20^ However, in neurosurgery, divorce rates do not follow this trend. A 2022 study of partners of neurosurgical residents reported a survey in which 18% responded they had considered termination of their relationship before the end of training, conversely 84% of respondents reported satisfaction in their relationship.^21^ Furthermore, the 2022 study found that most partner respondents would choose to be in a relationship with a neurosurgeon again.^21^

Despite relatively lower divorce rates among neurosurgeons compared with other surgical specialties, the effects of divorce on work for neurosurgeons is still profound. The financial implications of divorce including prenuptial agreements, alimony, and child support may significantly sway how neurosurgeons view their work, relative value unit targets, and their interest in teaching or research which are not associated with similar financial enticements as clinical work. Similarly, the requirement for meetings with attorneys and divorce hearings takes up time at work. The emotional drain of losing a relationship and the childcare responsibilities leave people re-evaluating themselves and their role as someone who heals and helps.

### Death of Loved One

Some of the most meaningful conversation during the panel discussion was when we discussed loss of a close family member. Our panelists shared experiences with death of a sibling, having to say goodbye to parent, and, most devastatingly, loss of a child. Some of these losses were after struggling with an illness and others were sudden and unexpected. The panelists noted feelings of being numb, inability to find meaning at work immediately after the death. They also noted that community support, spirituality, ability to share their feelings, and cry helped them weather the storms. The discourse involved significant discussion about suicide.

In recent years, rates of suicide have dramatically increased and is now one of the leading causes of death among adolescents.^22^ Suicide is a reality that neurosurgeons encounter among patients, family members, and friends and that they struggle with personally. We often encounter patients with self-inflicted gunshot wounds to the head or spine injuries from attempted hanging. Moreover, the panelists at the CNS special session discussed losing their own child or sibling to suicide. The shock and grief from discovering a loved one whose suffering has led to self-harm is unlike other types of grief. In a limited survey of practicing neurosurgeons, respondents discussed the feelings of feeling fragility after these losses. They also discussed that “mental health” is just health, and this approach would remove the stigma. Interestingly, one of the respondents noted that their support really came from their friends and community, not from the institution. However, when a medical student later experienced something similar, the outpouring of support from the institution was dramatic and in stark contrast with what faculty and staff are afforded. Sharing experiences such as these provide the opportunity to identify gaps in our systems for improvement in how we support one another.

Within the surgical profession, higher rates of suicide are being observed compared with other specialties.^23^ In a study published in 2010, investigators evaluated mortality rates among neurosurgeons between 1979 and 2005. They observed a lower proportion of neurosurgeons with death by suicide than predicted.^24^ Instead, leading causes of reported deaths in neurosurgeons were cancer and heart disease.^24^ However, these data are likely confounded by the stigma associated with suicide and death, often being attributed to another cause to avoid the uncomfortable discussions that family and friends may be trying to avoid. For example, one of the speakers in our panel noted personally knowing 2 neurosurgeons who took their own lives within the last 5 years. The cause of death reported at their funerals was a cardiac event. Increased suicide risk may be attributable to high rates of burnout, depression, intense workloads in high-stakes environments, and cultural or institutional norms that provide little space for vulnerability and cultivation of health.^25^

These challenges stress the profound importance of community in navigating grief. A growing body of evidence suggests that effective bereavement counseling and general social support can be a critical component of the healing process.^26,27^ The strength of connection, whether through faith, friendship, or shared experience, can serve as a life-saving force for those left behind.

### The Unspoken Challenges in Neurosurgery

The culture within neurosurgery, with its focus on precision and perfection, can sometimes lead to reluctance in acknowledging vulnerability or seeking help in the aforementioned challenges of personal and professional life, including mis-aligned work incentives, divorce, family planning, disability, and death (Figure).

**FIGURE.**
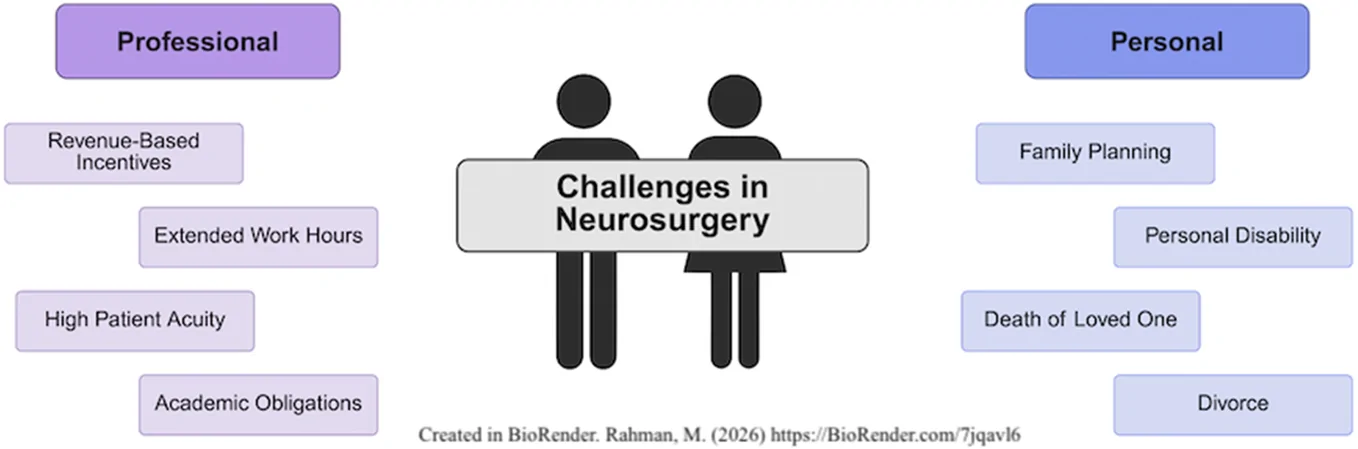
Personal and professional challenges experienced in the neurosurgical profession. *Created in BioRender. Rahman, M. (2026)* https://BioRender.com/7jqavl6.

The common themes during the panel discussion revolved around initial feelings of loneliness, isolation from colleagues in neurosurgery, numbness, extreme grief, and fear. The neurosurgical profession is replete with images of saving lives, strength, and confidence. Neurosurgeons tend to exude these qualities with patients, with colleagues, and, in interactions with community. This general stance is not conducive to vulnerability.^28^

Our panelists discussed the strategies that helped them out of the darkness which included leaning on others for their strength during the time of weakness, spirituality to make meaning of suffering, and community activities. They also shared the importance of work as a stabilizing force and ultimately how personal tragedy often helped them rediscover to meaning of being a neurosurgeon. In his personal account of surviving exile in Siberia as a child and experiencing family members dying of starvation, Andrew Bienkowski notes in his book One Life to Give: “Of course, we do not look forward to the difficult times in our lives. We do not welcome suffering. But radical gratitude is not experienced in the midst of suffering; it is only recognizable in hindsight. Once we look back at the difficult times, appreciate how far we've come, and admire where we are today, only then are we open to the healing that radical gratitude offers.” This theme was evident in the discussions with our panelists who expressed darkness and lessons that could only be articulated now that they have overcome and found stable ground during a tumultuous time.

The learning from these hardships is incredibly important for the development of neurosurgeons. Ultimately these lessons have a profound impact on the ability to be empathetic and skilled neurosurgeons who can help patients navigate the often heart-breaking journey of having a neurosurgical disease. Sharing these lessons within the profession in existing forums for how to clip an aneurysm, how to place a pedicle screw, and how to train a resident will allow neurosurgeons to lead both within and outside of our field of neurosurgery.

## LESSONS LEARNED

Key lessons emerged during the panel discussion. These included:

- Grief is common but rarely acknowledged in neurosurgical culture
- Personal loss often manifests as perceived performance decline before emotional recognition
- Community and peer support are more impactful than institutional response
- Identity disruption is a central component of physician grief

Actional recommendations for individuals include distinction of grief from burnout and engaging in peer support and structured reflection. Institutions may support physicians with formal bereavement and crisis accommodations and confidential mental health access, which is increasingly accessible. Incorporation of grief into wellness curricula or educational offerings will normalize discussion of grief in the specialty and create forums for neurosurgeons to support each other through significant loss. By replacing the expectation of silent trauma absorption with structured avenues for processing loss, neurosurgeons can redefine resilience as a collective, sustainable practice rather than an individual burden.

## CONCLUSION

Grief is an inevitable but under-recognized component of a career in neurosurgery. By reframing personal and professional adversity through the lens of loss, we can better understand the emotional experiences of neurosurgeons and develop more effective support systems. Distinguishing grief from burnout is essential not only for conceptual clarity but also for designing meaningful interventions. Cultural change within neurosurgery will require both individual vulnerability and institutional accountability. Recognizing and addressing grief is not a departure from excellence but rather a necessary step toward sustaining it.
